# Host phenology can drive the evolution of intermediate virulence strategies in some obligate‐killer parasites

**DOI:** 10.1111/evo.14507

**Published:** 2022-05-07

**Authors:** Hannelore MacDonald, Erol Akçay, Dustin Brisson

**Affiliations:** ^1^ Department of Biology University of Pennsylvania Philadelphia Pennsylvania USA

**Keywords:** adaptation, models/simulations, parasitism, trade‐offs

## Abstract

Traditional mechanistic trade‐offs between transmission and virulence are the foundation of nearly all theory on parasite virulence evolution. For obligate‐host killer parasites, evolution toward intermediate virulence depends on a trade‐off between virulence (time to death) and transmission (the number of progeny released upon death). Although several ecological factors impact optimal virulence strategies constrained by trade‐offs, these factors have been insufficient to explain the intermediate virulence levels observed in nature. The timing of seasonal activity, or phenology, is a factor that commonly influences ecological interactions but is difficult to incorporate into virulence evolution studies. We present a mathematical model of a seasonal obligate‐killer parasite to study the impact of host phenology on virulence evolution. The model demonstrates that host phenology can select for intermediate parasite virulence even when a traditional mechanistic trade‐off between transmission and virulence is omitted. The optimal virulence strategy is impacted by both the host activity period duration and the host emergence timing variation. Parasites with lower virulence strategies are favored in environments with longer host activity periods and when hosts emerge synchronously. The results demonstrate that host phenology can be sufficient to select for intermediate virulence strategies, providing an alternative driver of virulence evolution in some natural systems.

The evolutionary causes and consequences of parasite virulence remain enigmatic despite decades of research. It was once thought that parasites evolve ever lower levels of virulence to preserve their primary resource for future parasite generations (Smith, 1904). However, natural selection favors traits that improve short‐term evolutionary fitness even if those traits negatively impact the environment for future generations (Hamilton, 1964a, 1964b). The key breakthrough that propels virulence evolution research to this day assumes that within‐host mechanistic trade‐offs define parasite virulence strategies. Classic mechanistic trade‐offs between transmission and virulence are defined as a positive correlation between the number of parasites released from an infected host and host morbidity or mortality such that the transmission rate cannot increase without a correlated increase in virulence due to biological, biochemical, or physical constraints (Cressler et al., 2016; Alizon et al., 2009). In the absence of a mechanistic trade‐off that assumes the number of infectious progeny released upon host death (transmission) increases with the time between infection and parasite‐induced host death (virulence), obligate‐host killer parasites maximize their fitness by immediately killing their hosts in order to infect naïve hosts (Levin and Lenski, 1983). The diversity of intermediate virulence strategies among obligate‐host killer parasites has been explained exclusively by assuming a mechanistic trade‐off between transmission rate and virulence (Ben‐Ami, 2017; Caraco and Ing Wang, 2008; Ebert and Weisser, 1997; Jensen et al., 2006; Levin and Lenski, 1983; Wang, 2006).

Many ecological factors and environmental conditions have been shown to alter the optimal virulence strategies driven by mechanistic trade‐offs within models. For example, it is well established that varying environmental conditions, such as the extrinsic host death rate, often shift the optimal virulence strategy governed by a mechanistic trade‐off (Anderson and May, 1982; Cooper et al., 2002; Gandon et al., 2001; Lenski and May, 1994). However, no environmental condition, *in the absence* of an explicitly modeled correlation between virulence and transmission, has been shown to select for intermediate virulence.

The timing of seasonal activity, or phenology, is an environmental condition affecting all aspects of life cycles, including reproduction, migration, and diapause, in most species (Anderson et al., 2012; Elzinga et al., 2007; Forrest and Miller‐Rushing, 2010; Lustenhouwer et al., 2018; Novy et al., 2013; Pau et al., 2011; Park, 2019). The phenology of host species also impacts the timing and prevalence of transmission opportunities for parasites, which could alter optimal virulence strategies (Altizer et al., 2006; Biere and Honders, 1996; Gethings et al., 2015; Hamer et al., 2012; Martinez, 2018; McDevitt‐Galles et al., 2020; MacDonald et al., 2020; Ogden et al., 2018). For example, host phenological patterns that extend the time between infection and transmission are expected to select for lower virulence, as observed in some malaria parasites (*Plasmodium vivax*). In this system, high‐virulence strains persist in regions where mosquitoes are present year‐round, whereas low‐virulence strains are more common in regions where mosquitoes are nearly absent during the dry season (White et al., 2016). Although host phenology likely impacts virulence evolution in parasites (Donnelly et al., 2013; King et al., 2009; Sorrell et al., 2009; van den Berg et al., 2011), it remains unclear whether this environmental condition can have a sufficiently large impact to select for an intermediate virulence phenotype in the absence of a mechanistic trade‐off.

Here, we investigate the impact of host phenology on the virulence evolution of an obligate‐killer parasite. Our model assumes no within‐host mechanistic trade‐off, defined as the within‐host mechanistic link between transmission and virulence such that the transmission rate (infectious parasites released per infected individual) cannot increase without a correlated decrease in virulence (increased time between infection and parasite‐induced host death) (Alizon et al., 2009; Cressler et al., 2016). We demonstrate that intermediate virulence is adaptive when host activity patterns are highly seasonal, establishing that environmental context alone is sufficient to drive the evolution of intermediate virulence in disease systems that conform to the assumptions of the model. Further, multiple features of host seasonal activity, including season length and the synchronicity at which hosts first become active during the season, impact the optimal virulence level of parasites. These results provide an alternative framework that can account for virulence evolution in some natural systems.

## MODEL DESCRIPTION

**Figure 1:**
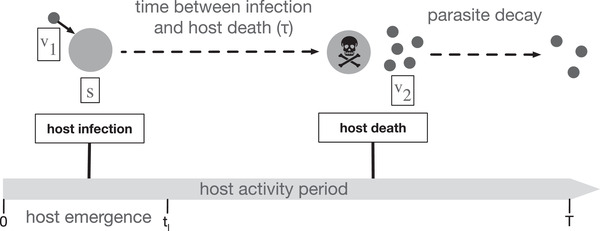
*Infection diagram*: The host cohort, $\hat{s}$, emerges from time t=0 to $t=t_{l}$, all *v*_1_ parasites emerge at t=0. Hosts do not reproduce during the season. Infections generally occur early in the season when host density is high. Parasite‐induced host death occurs after time τ, at which point new parasites, *v*_2_, are released. *v*_2_ decays in the environment from exposure. Parasites only have time to complete one round of infection per season. *v*_2_ parasites in the environment at t=T will carryover and emerge at the beginning of the next season

The model describes the transmission dynamics of a free‐living, obligate‐killer parasite that infects a seasonally available host (Fig. 1) in the presence and absence of a within‐host, mechanistic link between transmission and virulence. The host cohort, $\hat{s}$, enters the system at the beginning of the season over a period given by the function $g(t,t_{l})$. Hosts, *s*, have nonoverlapping generations and are alive for one season. The parasite, *v*, infects hosts while they are briefly susceptible early in their development (e.g., univoltine insects parasitized by ichneumonids; Delucchi, 1982; Kenis and Hilszczanski, 2007). The parasite must kill the host to release new infectious progeny. The parasite completes one round of infection per season because the incubation period of the parasite is longer than the duration of time the host spends in the susceptible developmental stage. This transmission scenario occurs in nature if all susceptible host stages emerge over a short period of time each season so that there are no susceptible host stages available when the parasite eventually kills its host. Parasites may also effectively complete only one round of infection per season if the second generation of parasites does not have enough time in the season to complete its life cycle in the short‐lived host.

We ignore the progression of the susceptible stage, *s*, to later life stages as it does not impact transmission dynamics. To keep track of these dynamics, we refer to the generation of parasites that infects hosts in the beginning of the season as *v*_1_ and the generation of parasites released from infected hosts upon parasite‐induced death as *v*_2_. τ is the delay between host infection by *v*_1_ and host death when *v*_2_ are released. τ is equivalent to virulence where low‐virulence parasites have long τ and high‐virulence parasites have short τ. The initial conditions in the beginning of the season are $s\left(0\right)=0,v_{1}\left(0^{+}\right)=v_{2}\left(0^{-}\right),v_{2}\left(\tau \right)=0$. The transmission dynamics in season *n* are given by the following system of delay differential equations (all parameters are described in Table 1):

(1a) $$\begin{array}{cc} & \frac{ds}{dt}=\hat{s}g\left(t,t_{l}\right)-\mu s\left(t\right)-\alpha s\left(t\right)v_{1}\left(t\right), \end{array}$$

(1b) $$\begin{array}{cc} & \frac{dv_{1}}{dt}=-\delta v_{1}\left(t\right), \end{array}$$

(1c) $$\begin{array}{cc} & \frac{dv_{2}}{dt}=\alpha \beta e^{-\mu \tau }s\left(t-\tau \right)v_{1}\left(t-\tau \right)-\delta v_{2}\left(t\right), \end{array}$$

**Table 1:** Model parameters and their respective values

Parameter	Description	Value
*s*	Susceptible hosts	State variable
*v*_1_	Parasites that infect hosts in current season	State variable
*v*_2_	Parasites released in current season	State variable
$t_{l}$	Length of host emergence period	Time (varies)
*T*	Season length	Time (varies)
$\hat{s}$	Emerging host cohort size	10^8^ hosts
α	Transmission rate	10^−8^/(parasite × time)
β	Number of parasites produced upon host death	Parasites (varies)
δ	Parasite decay rate in the environment	2 Parasites/parasite/time
μ	Host death rate	0.5 Hosts/host/time
τ	Time between host infection and host death (1/virulence)	Time (evolves)

where μ is the host death rate, δ is the decay rate of parasites in the environment, α is the transmission rate, and β is the number of parasites produced upon host death. We consider scenarios where there is and is not an explicit link between β (transmission) and τ (1/virulence). We make the common assumption for free‐living parasites that the removal of parasites through transmission (α) is negligible (Anderson and May, 1981; Dwyer, 1994), that is, (1b) ignores the term $-\alpha s\left(t\right)v_{1}\left(t\right)$.

The function $g(t,t_{l})$ captures the per‐capita host emergence rate by specifying the timing and length of host emergence. We use a uniform distribution (U(•)) for analytical tractability, but other distributions can be used:

 

$$g\left(t,t_{l}\right)=\left\{\begin{array}{cc} \frac{1}{t_{l}} & 0\leq t\leq t_{l}\\ 0 & t_{l}<t\leq T \end{array}\right.$$

where $t_{l}$ denotes the length of the host emergence period and *T* denotes the season length. The season begins ($t_{0}=0$) with the emergence of the susceptible host cohort, $\hat{s}$. The host cohort emerges from $0\leq t\leq t_{l}$. *v*_2_ parasites remaining in the system at t=T give rise to next season's initial parasite population ($\hat{v}=v_{1}\left(0\right)$). Parasites that have not killed their host by the end of the season do not release progeny. Background mortality arises from predation or some other natural cause. We assume that infected hosts that die from background mortality do not release parasites because the parasites are either consumed or the latency period corresponds to the time necessary to develop viable progeny (Wang, 2006; White, 2011). We ignore the impact of infection for host demography and assume $\hat{s}$ is constant each year (e.g., a system where host regulation by parasites is negligible). We solve Equations (1a)– (1c) analytically in Appendix A.

### PARASITE FITNESS

A parasite introduced into a naïve host population persists or goes extinct depending on the length of the host emergence period and season length. The stability of the parasite‐free equilibrium is determined by the production of *v*_2_ resulting from infection of *s* given by

$$\begin{array}{ccc} & & v_{2}\left(T\right)=e^{-\delta (T-t_{l}-\tau )}\\ & & \left(v_{2}\left(t_{l}\right)+\alpha \beta e^{-\mu \tau }\hat{v}s\left(t_{l}\right)\int_{0}^{T-t_{l}-\tau }e^{-\frac{\alpha \hat{v}e^{-\delta (u+t_{l})}\left(-1+e^{\delta u}\right)}{\delta }-\delta t_{l}-\mu u}du\right) \end{array}$$

when $\tau <T-t_{l}$ and by

$$\begin{array}{cc} & v_{2}\left(T\right)=\frac{\alpha \beta e^{-\mu \tau }\hat{v}\hat{s}}{t_{l}}e^{-\delta (T-\tau )}\int_{0}^{T-\tau }e^{\left(-\mu u+\frac{\alpha \hat{v}e^{-\delta u}}{\delta }\right)}\int_{0}^{u}e^{\left(\mu x-\frac{\alpha \hat{v}e^{-\delta x}}{\delta }\right)}dxdu \end{array}$$

when $\tau >T-t_{l}$.

The parasite‐free equilibrium will be unstable and a single parasite introduced into the system at the beginning of the season will persist if the density of *v*_2_ produced by time *T* is greater than or equal to $\hat{v}=v_{1}\left(0\right)=1$ (*i.e*. $v_{2}\left(T\right)\geq 1$, modulus is greater than unity). This means that each parasite infecting a host produces more than one parasite on average. See Appendix A for details of the analytical solution.

### PARASITE EVOLUTION

To study how parasite traits adapt given different seasonal host activity patterns, we use evolutionary invasion analysis (Geritz et al., 1998; Metz et al., 1992). We first extend system (1) to follow the invasion dynamics a rare mutant parasite:

(2a) $$\begin{array}{cc} & \frac{ds}{dt}=\hat{s}g\left(t,t_{l}\right)-\mu s\left(t\right)-\alpha s\left(t\right)v_{1}\left(t\right)-\alpha_{m}s\left(t\right)v_{1m}\left(t\right), \end{array}$$

(2b) $$\begin{array}{cc} & \frac{dv_{1}}{dt}=-\delta v_{1}\left(t\right), \end{array}$$

(2c) $$\begin{array}{cc} & \frac{dv_{1m}}{dt}=-\delta_{m}v_{1m}\left(t\right), \end{array}$$

(2d) $$\begin{array}{cc} & \frac{dv_{2}}{dt}=\alpha \beta e^{-\mu \tau }s\left(t-\tau \right)v_{1}\left(t-\tau \right)-\delta v_{2}\left(t\right), \end{array}$$

(2e) $$\begin{array}{cc} & \frac{dv_{2m}}{dt}=\alpha_{m}\beta_{m}e^{-\mu \tau_{m}}s\left(t-\tau_{m}\right)v_{1m}\left(t-\tau_{m}\right)-\delta_{m}v_{2m}\left(t\right), \end{array}$$

where subscript *m* refers to the invading mutant parasite and its corresponding traits. See Appendix B for details of the time‐dependent solutions for Equations (2a)– (2e).

The invasion fitness of a rare mutant parasite depends on the density of $v_{2m}$ produced by the end of the season ($v_{2m}\left(T\right)$) in the environment set by the resident parasite at equilibrium density ${\hat{v}}^{*}$. The mutant parasite invades in a given host phenological scenario if the density of $v_{2m}$ produced by time *T* is greater than or equal to the initial $v_{1m}\left(0\right)=1$ introduced at the start of the season ($v_{2m}\left(T\right)\geq 1$). When $\tau <T-t_{l}$, mutant invasion fitness can be found using

(3a) $$\begin{array}{cc} v_{2m}\left(T\right)= & e^{-\delta_{m}\left(T-t_{l}-\tau_{m}\right)}(v_{2m}\left(t_{l}\right)+\alpha_{m}\beta_{m}e^{-\mu \tau_{m}}v_{1m}\left(0\right)s\left(t_{l}\right)\\ & \int_{0}^{T-t_{l}-\tau_{m}}e^{-\frac{\alpha_{m}v_{1m}\left(0\right)e^{-\delta_{m}\left(u+t_{l}\right)}\left(-1+e^{\delta_{m}u}\right)}{\delta_{m}}-\frac{\alpha {\hat{v}}^{*}e^{-\delta (u+t_{l})}\left(-1+e^{\delta u}\right)}{\delta }-\delta_{m}t_{l}-\mu u}du). \end{array}$$

When $\tau >T-t_{l}$, mutant invasion fitness can be found using

(3b) $$\begin{array}{cc} v_{2m}\left(T\right)= & \frac{\alpha_{m}\beta_{m}e^{-\mu \tau_{m}}v_{1m}\left(0\right)\hat{s}}{t_{l}}e^{-\delta_{m}\left(T-\tau_{m}\right)}\\ & \int_{0}^{T-\tau_{m}}e^{\left(-\mu u+\frac{\alpha {\hat{v}}^{*}e^{-\delta u}}{\delta }+\frac{\alpha_{m}v_{1m}\left(0\right)e^{-\delta_{m}u}}{\delta_{m}}\right)}\\ & \int_{0}^{u}e^{\left(\mu x-\frac{\alpha {\hat{v}}^{*}e^{-\delta s}}{\delta }-\frac{\alpha_{m}v_{1m}\left(0\right)e^{-\delta_{m}x}}{\delta_{m}}\right)}dxdu. \end{array}$$

To study the evolution of virulence traits, we first assume all other resident and mutant traits are identical (e.g., $\alpha =\alpha_{m}$). Note that when there is no trade‐off between β and τ, the parasite growth rate in the host is essentially the trait under selection. That is, β is constant regardless of τ, thus the trait that is effectively evolving is the rate that new parasites are assembled in between infection and host death (e.g., long τ corresponds to slow assembly of new parasites.) To find optimal virulence for a given host phenological scenario, we find the uninvadable trait value that maximizes (3). That is, the virulence trait, $\tau^{*}$, that satisfies

(4a) $$\begin{array}{cc} & \frac{\partial v_{2m}\left(T\right)}{\partial \tau_{m}}{|}_{\tau_{m}=\tau_{r}}=0 \end{array}$$

(4b) $$\begin{array}{cc} & \frac{\partial^{2}v_{2m}\left(T\right)}{\partial \tau_{m}^{2}}{|}_{\tau_{m}=\tau_{r}}<0. \end{array}$$

Note that the measure in Equations (3a) and (3b) incorporates the effect of the resident on the population state (the number of susceptibles over one season), which means that it is not a measure of *R*_0_ (which by definition assumes a nondisease environment). Thus, we can use $v_{2m}\left(T\right)$ as defined in (3a) and (3b) as a maximand in evolutionary dynamics (Lion and Metz, 2018).

To study the impact of mechanistic trade‐offs between transmission and virulence on virulence evolution, we assume that the number of parasites produced at host death is a function of the time between infection and host death (β(τ)). For example, mutant invasion fitness for $\tau <T-t_{l}$ can be found using

(5) $$\begin{array}{cc} v_{2m}\left(T\right)= & e^{-\delta_{m}\left(T-t_{l}-\tau_{m}\right)}(v_{2m}\left(t_{l}\right)+\alpha_{m}\beta \left(\tau_{m}\right)e^{-\mu \tau_{m}}v_{1m}\left(0\right)s\left(t_{l}\right)\\ & \int_{0}^{T-t_{l}-\tau_{m}}e^{-\frac{\alpha_{m}v_{1m}\left(0\right)e^{-\delta_{m}\left(u+t_{l}\right)}\left(-1+e^{\delta_{m}u}\right)}{\delta_{m}}-\frac{\alpha {\hat{v}}^{*}e^{-\delta (u+t_{l})}\left(-1+e^{\delta u}\right)}{\delta }-\delta_{m}t_{l}-\mu u}du). \end{array}$$

We then find $\tau^{*}$ that satisfies (4a) and (4b) using Equation (5).

## RESULTS

Host phenology is sufficient to drive the evolution of intermediate virulence in systems that conform to the assumptions of the model. Host phenology is composed of the duration of the activity period and the distribution of initial emergence times, both of which impact the optimal parasite virulence level. Temporally constrained host activity periods within each season can select against both extremely high and extremely low virulence levels resulting in an intermediate optimal level of virulence. Low virulence is selected against as parasites that do not kill the infected host prior to the end of the host activity period fail to produce progeny and thus have no evolutionary fitness. By contrast, highly virulent parasites kill their hosts quickly and the released progeny decay in the environment for the remainder of the activity period. Thus, progeny released early in the host activity period are more likely to die in the environment prior to contacting a naïve host in the following season. An intermediate virulence level that allows parasites to kill their host prior to the end of the activity period, but not so quickly that the progeny produced are likely to decay in the environment, result in the greatest evolutionary fitness.

**Figure 2:**
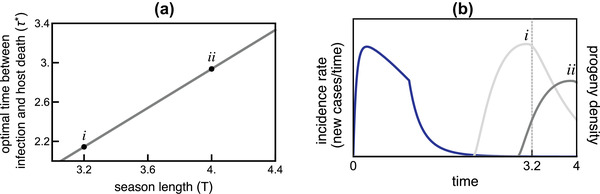
Host seasonality is sufficient to select an intermediate virulence strategy. (a) The temporal duration between infection and host death ($\tau^{*}$) always evolves to a value that is greater than 0 (extreme virulence) and less than the season length (extremely low virulence); the intermediate virulence strategy maximizes parasite fitness in environments where host activity is seasonal. The optimal parasite‐induced host death rate results in host death and progeny release shortly before the end of the season (t=T). Progeny release just prior to the end of the season limits progeny decay from environmental exposure while avoiding progeny dying within their host at the end of the season. Points (i) and (ii) are representative examples of optimal virulence strategies in environments with shorter (T=3.2) or longer (T=4) host activity periods, respectively. $\tau^{*}$ is found using Equation (4a) when there is no trade‐off between transmission and virulence. (b) Higher parasite virulence is favored in environments with limited host activity periods. Parasites with greater virulence produce more progeny that survive to the end of the season when seasons are short. That is, the density of the more virulent progeny (i) at T=3.2 is greater than the density of the less virulent progeny (ii). The more virulent parasites kill their hosts quickly such that few infected hosts survive to the end of the season and the progeny released spend little time in the environment. By contrast, less virulent parasites (ii) often fail to kill their hosts and release progeny prior to the end of short activity periods (T=3.2). Longer seasons (T=4) favor less virulent parasites (ii) as they kill their hosts closer to the end of the season such that fewer of their released progeny decay in the environment (ii) than the progeny of the more virulent parasites that are released earlier in the season (i). The blue line represents the incidence rate of new infections; $t_{l}=1$; all other parameters found in Table 1

The optimal virulence level increases linearly with decreases in the duration of host activity (Fig. 2). Virulent parasites in environments where host activity periods are short minimize the cost of not producing progeny from infected hosts and do not incur the costs of progeny decaying in the environment. By contrast, environments where host activity periods are long favor parasites with a long incubation period to limit the cost of progeny decay due to environmental exposure while still killing hosts prior to the end of the season. The optimal level of virulence in all environmental scenarios results in parasite‐induced host death just prior to the end of the seasonal activity period. The linear increase in optimal virulence as season length decreases suggests that parasite fitness is optimized when host death occurs at a fixed time before the end of the season.

**Figure 3:**
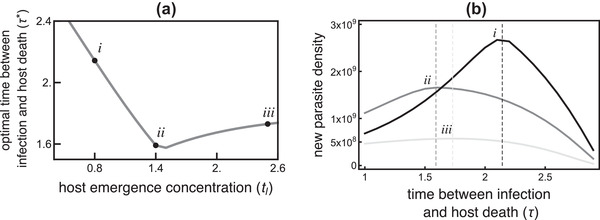
The variation in host emergence timing impacts the optimal virulence strategy. (a) Parasites with lower virulence are favored in environments where nearly all hosts emerge simultaneously (i). Progeny from the low‐virulence parasites are released nearly simultaneously just prior to the end of the season. High‐virulence parasites are favored in environments where host emergence period length is moderate (ii). Moderate variation in host emergence decreases the average time between infection and the end of the season and favors parasites with a high‐virulence strategy such that few infected hosts survive to the end of the season. Parasites in environments where host emergence variation is very high maximize the number of progeny that survive to the next season using a moderate virulence strategy (iii). Parasites in these environments suffer the costs of hosts that are infected later in the season not releasing progeny as well as progeny decay in the environment when released from early‐infected hosts. A moderate virulence strategy allows hosts infected around the mid‐season peak in incidence to release progeny while limiting the decay of these progeny. $\tau^{*}$ is found using Equation (4a) when there is no trade‐off between transmission and virulence. (b) Equilibrium density of parasites with the optimal virulence strategy for their environment decreases with increasing variation in host emergence timing. Optimal virulence results in peak equilibrium in new parasites density, indicated by the vertical lines. T=3; other parameters found in Table 1

Variation in the time at which each host first becomes active during the activity period also impacts the virulence levels that maximize parasite fitness (Fig. 3). Synchronous host emergence results in a rapid and early spike in infection incidence due to the simultaneous availability of susceptible hosts and the abundance of free parasites. The long duration between host infection and the end of the activity period favors low‐virulence parasites that kill their host near the end of the season (Fig. 3a, i). Variability in the time at which each susceptible host initially becomes active decreases the average time between infection and the end of the season, thus favoring more virulent parasites (Fig. 3a, ii). That is, the large proportion of infections that occur later in the season require higher virulence to be able to release progeny before the activity period ends. This higher virulence level comes at the cost of progeny from hosts infected early in the season decaying in the environment. Thus, the number of progeny that survive to the next season decreases with increasing variation in host emergence times (Fig. 3b).

High variability in host emergence timing results in an optimal virulence strategy that is much greater than in environments with synchronous host emergence, but lower than in environments with a moderate distribution (Fig. 3). That is, increasing variation in host emergence timing favors parasites with higher virulence, but only when variation in host emergence timing is moderate. In environments where the variation in host emergence timing is high, increasing variation in host emergence timing favors parasites with slightly lower virulence (Fig. 3a, iii). Lower virulence is favored in high‐emergence variability environments because the number of new infections occurring late in the season, where high virulence would be advantageous, are relatively rare due to small parasite population sizes at the beginning of the season and parasite decay during the season. Initial parasite population sizes are smaller in environments with broadly distributed host emergence timing as fewer total hosts are infected because infection probability is density dependent, and thus fewer progeny are produced. Most parasites that find a susceptible host do so early in the season resulting in additional decreases to the already small parasite population size. The optimal virulence strategy allows parasites that infect hosts around the peak of new infections—occurring mid‐season when susceptible host densities are greatest and parasite populations have not decayed substantially—to release progeny while limiting decay of these progeny. Parasites in environments where the distribution in host emergence times is very broad suffer the costs of both decay of the progeny released by early‐infected hosts and the cost of late infected hosts not releasing progeny, collectively causing these environments to maintain low densities of moderately virulent pathogens (Fig. 3b, iii).

**Figure 4:**
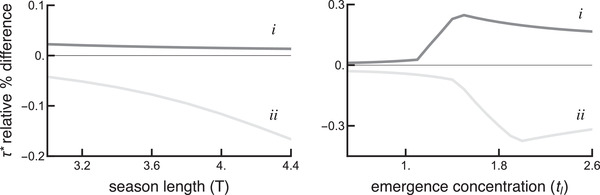
Mechanistic transmission‐virulence trade‐offs shift the optimal virulence strategy but are not necessary to favor intermediate virulence in environments with seasonal host activity. The optimal virulence level for parasites in which longer durations of infection result in *more* progeny is slightly lower than for parasites that are not constrained with this mechanistic trade‐off in the same environment (i). This mechanistic trade‐off elevates the fitness benefit of longer duration infections by compensating for the cost of infected hosts dying without releasing progeny. The optimal virulence level for parasites in which longer infection durations result in *fewer* progeny is greater than for parasites without this trade‐off in the same seasonal environments (ii). This mechanistic trade‐off elevates the fitness benefit of shorter duration infections despite the cost of greater progeny decay in the environment. $\tau^{*}$ was found using Equation (4a) when there is no trade‐off between transmission and virulence and then compared to $\tau^{*}$ constrained by a trade‐off with transmission. Trade‐off for i:β(τ)=99(τ+0.5), trade‐off for ii:β(τ)=99(−τ+4). All other parameters found in Table 1

Mechanistic virulence–transmission trade‐offs can modify the optimal virulence strategy in seasonal environments but are not necessary for natural selection to favor intermediate virulence phenotypes. The optimal virulence strategy is slightly lower in models that include a trade‐off where duration of infection is positively correlated with progeny production than in models with the same phenological parameters that do not include the trade‐off (Fig. 4). Including this trade‐off increases the fitness benefit of longer duration infections to a greater extent than the costs associated with infected host mortality not caused by the parasite. By contrast, the optimal virulence strategy is greater in models that include a trade‐off where duration of infection is negatively correlated with progeny production than in similar models without the trade‐off (Fig. 4). Including this trade‐off increases the fitness benefit of shorter duration infections despite the added costs of greater parasite decay due to environmental exposure. Including mechanistic trade‐offs modifies the selection pressures on virulence strategies but are not essential for an intermediate virulence strategy to be optimal in seasonal environments.

## DISCUSSION

Nearly all theory developed to explain parasite virulence evolution has utilized mechanistic trade‐offs between virulence and other traits important to parasite fitness (Alizon et al., 2009; Cressler et al., 2016). The results of this study show that seasonal host activity, in the absence of an assumed positive correlation between virulence and transmission, can account for the evolution of intermediate virulence in some specific situations. Both aspects of phenology, the duration of the host activity period and host emergence synchronicity, impact the virulence strategy that maximizes the evolutionary fitness of parasites. Although mechanistic trade‐offs between virulence and transmission can shift the optimal virulence level as predicted by prior theory, these trade‐offs are not essential for intermediate virulence to evolve in this system. The current demonstration that an ecological context is sufficient to select for intermediate virulence broadens the scope of factors that can explain the diversity of parasite virulence strategies. Thus, the evolution of intermediate virulence in natural systems may be governed by a mechanistic trade‐off or by ecological factors in some systems.

Seasonal host activity can select for intermediate virulence by generating conflicting costs for releasing progeny too early or too late in the season. Low virulence is maladaptive for parasites in this system as they do not kill their host before the end of the season and create no progeny. High virulence is also maladaptive as progeny released early are more likely to die due to environmental exposure. The conflicting costs of not releasing progeny before the end of the season and releasing progeny too early in the season selects for intermediate virulence levels. Optimal virulence results in parasite‐induced host death and the release of progeny slightly before the end of the host activity period.

The result predicting adaptive evolution toward intermediate virulence stands in contrast to many prior theoretical investigations of obligate‐killer parasites. Prior models of obligate‐killer parasites predict ever‐increasing virulence in the absence of mechanistic trade‐offs (Caraco and Ing Wang, 2008; Ebert and Weisser, 1997; Levin and Lenski, 1983; Sasaki and Godfray, 1999). In simple obligate‐killer models, killing infected hosts as quickly as possible is expected to maximize fitness as the early release of progeny permits infection of additional susceptible hosts resulting in a rapid exponential increase of parasites in the system. To date, only mechanistic trade‐offs between virulence and transmission‐associated factors as well as development time constraints have been demonstrated to constrain maximal virulence in obligate‐killer parasite models (Ben‐Ami, 2017; Caraco and Ing Wang, 2008; Ebert and Weisser, 1997; Jensen et al., 2006; Wang, 2006). In contrast, our results indicate that host phenology can create conditions that favor intermediate virulence in obligate‐killer parasites even if a negative correlation between virulence and transmission is not included in the model. In the current model, intermediate virulence is favored as seasonal host absence increases the evolutionary benefit of remaining within hosts in order to reduce deaths in the free‐living stage caused by environmental exposure (Reece et al., 2017).

Variation in host emergence synchronicity impacts the optimal virulence strategy of parasites in this system. High parasite virulence is favored at low host emergence synchronicity. Low‐emergence synchronicity slows incidence by decreasing both the rate hosts emerge and parasite equilibrium density. When more infections occur later in the season, parasites have less time to release new parasites before the end of the season. High parasite virulence is adaptive because hosts have a low expected life span at the time of infection. This result is analogous to the prediction that high host mortality drives the evolution of high virulence (Anderson and May, 1982; Cooper et al., 2002; Gandon et al., 2001; Lenski and May, 1994). The timing of host activity can thus lead to the evolution of high virulence in a similar manner to how host demography impacts virulence.

The seasonal activity patterns of species with nonoverlapping generations may have large impacts on the virulence strategies of the parasites they host. For example, parasites and parasitoids of univoltine insects that complete one round of infection per host generation may maximize their fitness by releasing progeny just prior to the end of the season (Delucchi, 1982; Kenis and Hilszczanski, 2007). The theoretical expectations presented here can be tested empirically by measuring the virulence strategies of parasites across the natural diversity of phenological patterns observed over the geographical range of many insect species. Similarly, experiments could rigorously assess the impact of both season length and host emergence variability on the fitness of parasites with different levels of virulence.

The prediction that shorter host activity periods can drive greater virulence is comparable to how the virulence of different *Theileria parva* strains varies between regions. High within‐host densities permit a virulent *T. parva* strain to be reliably transmitted to feeding nymphal tick vectors shortly after being infected by the adult stage in regions where the activity patterns of the two tick life stages overlap (Norval et al., 1991; Ochanda et al., 1996; Randolph, 1999). In contrast, the virulent strain is absent in regions where nymphal and adult activity is asynchronous while a less virulent strain that persists in hosts longer is maintained (Norval et al., 1991; Randolph, 1999). Thus, the prediction that the length of the host activity period is inversely correlated with virulence coincides with empirical observations of the distribution of *T. parva* strains.

Several features of the current model can be altered to investigate more complex impacts of phenology on virulence evolution. For example, relaxing the assumption of a constant host population size may result in a feedback between parasite fitness and host demography with consequences for population dynamics (Hilker et al., 2020). Additionally, parasite virulence evolution may select for alternative host phenological patterns that in turn select for parasite traits with lower impacts on host fitness. This modeling framework could also be tailored to explain virulence evolution in other seasonal disease systems, such as *Lepidoptera*–baculovirus systems, by relaxing certain assumptions, for example, parasites are monocyclic, decay rate is exponentially distributed (Baltensweiler et al., 1977; Woods and Elkinton, 1987). We will extend the current model to address these questions in future studies.

The model presented applies to obligate‐killer parasites that complete one round of infection per season (monocyclic) in hosts that have nonoverlapping generations. Currently, there is no evidence that disease systems that violate these assumptions can select for intermediate virulence without including a mechanistic trade‐off. Nevertheless, several prior models that included both host seasonality and mechanistic trade‐offs found qualitatively similar results as those presented here despite relaxing one or more of the strict assumptions in this model (King et al., 2009; Sorrell et al., 2009; van den Berg et al., 2011), suggesting that phenology can have a large impact on virulence outcomes. For example, longer seasons or longer periods between seasons have been shown to select for lower virulence in polycyclic parasites in seasonal environments (Sorrell et al., 2009; van den Berg et al., 2011), similar to the results presented here. Similarly, explicitly modeling parasite growth rates within hosts, which underlie the correlation between virulence and instantaneous transmission rates, selects for intermediate virulence levels that maximize transmission rates during host activity periods (King et al., 2009). By contrast, assuming that virulence levels are mechanistically associated with host density results in selection for higher virulence in seasonal environments (Donnelly et al., 2013). Future studies incorporating one or more of these competing forces with environmental decay of progeny could be sufficient to select for intermediate virulence in the absence of an assumed mechanistic trade‐off.

Some of the strict model assumptions can likely be relaxed without altering the result that phenology can be sufficient to select for intermediate virulence strategies. Relaxing the obligate‐killer assumption may result in the same qualitative result that intermediate virulence is adaptive in some cases. For example, longer latency periods that result in progeny release near the end of the season would still be adaptive for parasites that reduce host fecundity or increase host death rate, even if there is no correlation between the virulence level and instantaneous or life‐time transmission. Longer latency periods are equivalent to lower virulence in this type of system as infected hosts have more time to reproduce and thus higher fitness. This extension is not expected to qualitatively alter the results if the parasite transmission period is short relative to the season length. Many parasite–host systems conform to the assumptions of this model extension such as monocyclic plant pathogens (e.g., soil‐borne plant pathogens, demicyclic rusts, postharvest diseases), and many diseases systems infecting univoltine insects (Crowell, 1934; Gaulin et al., 2007; Holuša and Lukášová, 2017; Hamelin et al., 2011; Zehret al., 1982).

The importance of parasite virulence to both host–parasite interactions and public health policy has resulted in a concentrated research effort on virulence evolution. Nearly all theoretical research to date has incorporated a mechanistic trade‐off between virulence and transmission rates or infection duration, a hypothesis that is still essential to explain the evolution of intermediate virulence in most disease systems. However, ecological factors such as seasonal host activity or spatial structuring provide alternative theoretical frameworks that may account for virulence strategies in some natural systems (Boots and Sasaki, 1999; Kerr et al., 2006). Future work that identifies and empirically validates ecological factors that influence virulence evolution would be useful for predicting outbreaks of highly virulent parasites.

## CONFLICT OF INTEREST

The authors declare that there is no conflict of interest.

## AUTHOR CONTRIBUTIONS

Hannelore MacDonald and Dustin Brisson conceived of the presented idea and developed the theoretical framework; Hannelore MacDonald conducted the mathematical analysis and performed numerical simulations; Erol Akçay supervised the mathematical analysis and numerical simulations. All authors wrote the manuscript and gave final approval for publication and agreed to be held accountable for the work performed therein.

Associate Editor: M. Boots

Handling Editor: T. Chapman

## Data Availability

Code is available on the Github repository: https://github.com/hanneloremac/Host‐phenology‐drives‐the‐evolution‐of‐intermediate‐parasite‐virulence

## Appendix A

In Appendix A, we find analytical solutions for Equations (1a)– (1c) from the main text to study parasite fitness given different host phenological patterns:

(A1a)
$$\begin{array}{cc} & \frac{ds}{dt}=\hat{s}g\left(t,t_{l}\right)-\mu s\left(t\right)-\alpha s\left(t\right)v_{1}\left(t\right), \end{array}$$

(A1b)
$$\begin{array}{cc} & \frac{dv_{1}}{dt}=-\delta v_{1}\left(t\right), \end{array}$$

(A1c)
$$\begin{array}{cc} & \frac{dv_{2}}{dt}=\alpha \beta e^{-\mu \tau }s\left(t-\tau \right)v_{1}\left(t-\tau \right)-\delta v_{2}\left(t\right), \end{array}$$

with initial conditions: $s\left(0\right)=0,v_{1}\left(0^{+}\right)=v_{2}\left(0^{-}\right),v_{2}\left(\tau \right)=0$.

Equations (A1a)– (A1c) are solved analytically by describing host emergence using a uniform distribution

$$g\left(t,t_{l}\right)=\left\{\begin{array}{cc} \frac{1}{t_{l}} & 0\leq t\leq t_{l}\\ 0 & t_{l}<t. \end{array}\right.$$

To solve the dynamics during the host's activity period, we first find the analytical solution for $v_{1}\left(t\right)$:

$$v_{1}\left(t\right)=\hat{v}e^{-\delta t}.$$

We then use $v_{1}\left(t\right)$ to find the time‐dependent solution for s(t). We can then plug the time‐dependent solution for s(t) to find the time‐dependent solution for $v_{2}\left(t\right)$. Only parasites that infect hosts from 0<t<T−τ have enough time to kill hosts and release progeny before the end of the season. For $\tau <T-t_{l}$, parasites that infect hosts during host emergence ($0<t\leq t_{l}$) have time to kill hosts and release progeny before the end of the season as well as some parasites that infect hosts after host emergence has ended ($t>t_{l}$). For $\tau >T-t_{l}$, only some parasites that infect hosts during host emergence ($0<t\leq t_{l}$) have time to kill hosts and release progeny before the end of the season. Thus, two separate solutions are required depending on whether τ is greater or less than $T-t_{l}$. We first consider the case where $\tau <T-t_{l}$:

$$\begin{array}{cc} & s\left(t\right)=\left\{\begin{array}{cc} \frac{\hat{s}}{t_{l}}e^{\left(-\mu t+\frac{\alpha \hat{v}e^{-\delta t}}{\delta }\right)}\int_{0}^{t}e^{\left(\mu u-\frac{\alpha \hat{v}e^{-\delta u}}{\delta }\right)}du & 0<t<t_{l}\\ s\left(t_{l}\right)e^{\left(-\mu \left(t-tl\right)-\frac{\alpha \hat{v}e^{-\delta (t+t_{l})}\left(-1+e^{\delta t}\right)}{\delta }\right)} & t_{l}\leq t<T \end{array}\right.\\ & v_{2}\left(t\right)=\left\{\begin{array}{cc} \frac{\alpha \beta e^{-\mu \tau }\hat{v}\hat{s}}{t_{l}}e^{-\delta (t-\tau )}\int_{0}^{t-\tau }e^{\left(-\mu u+\frac{\alpha \hat{v}e^{-\delta u}}{\delta }\right)}\int_{0}^{u}e^{\left(\mu x-\frac{\alpha \hat{v}e^{-\delta x}}{\delta }\right)}dxdu & \tau <t<t_{l}\\ e^{-\delta (t-t_{l}-\tau )}\left(v_{2}\left(t_{l}\right)+\alpha \beta e^{-\mu \tau }\hat{v}s\left(t_{l}\right)\int_{0}^{t-t_{l}-\tau }e^{-\frac{\alpha \hat{v}e^{-\delta (u+t_{l})}\left(-1+e^{\delta u}\right)}{\delta }-\delta t_{l}-\mu u}du\right) & t_{l}\leq t<T, \end{array}\right. \end{array}$$

where $s(t_{l})$ and $v_{2}\left(t_{l}\right)$ are the densities of *s* and *v*_2_, respectively, when the emergence period of *s* ends.

For $\tau >T-t_{l}$, only some of the parasites that infect hosts from $0<t<t_{l}$ have enough time to kill hosts and release progeny before the end of the season. $v_{2}\left(t\right)$ are thus only produced from infections that occurred from $0<t<t_{l}$. The solution for $v_{2}\left(t\right)$ in this case is

$$\begin{array}{cc} & v_{2}\left(t\right)=\frac{\alpha \beta e^{-\mu \tau }\hat{v}\hat{s}}{t_{l}}e^{-\delta (t-\tau )}\int_{0}^{t-\tau }e^{\left(-\mu u+\frac{\alpha \hat{v}e^{-\delta u}}{\delta }\right)}\\ & \int_{0}^{u}e^{\left(\mu x-\frac{\alpha \hat{v}e^{-\delta x}}{\delta }\right)}dxdu\;\tau <t<T. \end{array}$$

A parasite introduced into a naïve host population persists or goes extinct depending on the host emergence period length and season length. The stability of the parasite‐free equilibrium when $\tau <T-t_{l}$ is determined by the production of *v*_2_ resulting from infection of *s* given by

$$\begin{array}{cc} & v_{2}\left(T\right)=e^{-\delta (T-t_{l}-\tau )}(v_{2}\left(t_{l}\right)+\alpha \beta e^{-\mu \tau }\hat{v}s\left(t_{l}\right)\\ & \int_{0}^{T-t_{l}-\tau }e^{-\frac{\alpha \hat{v}e^{-\delta (u+t_{l})}\left(-1+e^{\delta u}\right)}{\delta }-\delta t_{l}-\mu u}du). \end{array}$$

When $\tau >T-t_{l}$, the stability of the parasite‐free equilibrium is determined by

$$\begin{array}{cc} & v_{2}\left(T\right)=\frac{\alpha \beta e^{-\mu \tau }\hat{v}\hat{s}}{t_{l}}e^{-\delta (T-\tau )}\int_{0}^{T-\tau }e^{\left(-\mu u+\frac{\alpha \hat{v}e^{-\delta u}}{\delta }\right)}\int_{0}^{u}e^{\left(\mu x-\frac{\alpha \hat{v}e^{-\delta x}}{\delta }\right)}dxdu. \end{array}$$

The parasite‐free equilibrium is unstable and the parasite will persist in the system if the density of *v*_2_ produced by time *T* is greater than or equal to $\hat{v}=v_{1}\left(0\right)=1$ introduced at the beginning of the activity period of *s* (i.e., $v_{2}\left(T\right)\geq 1$, modulus is greater than unity). This expression is a measure of a parasite's fitness when rare for different host phenological patterns given $\tau >T-t_{l}$.

## Appendix B

In Appendix B, we find analytical solutions for Equations (2a)– (2e) from the main text to study the evolution of parasite virulence given different host phenological patterns:

(B1a)
$$\begin{array}{cc} & \frac{ds}{dt}=\hat{s}g\left(t,t_{l}\right)-\mu s\left(t\right)-\alpha s\left(t\right)v_{1}\left(t\right)-\alpha_{m}s\left(t\right)v_{1m}\left(t\right), \end{array}$$

(B1b)
$$\begin{array}{cc} & \frac{dv_{1m}}{dt}=-\delta_{m}v_{1m}\left(t\right), \end{array}$$

(B1c)
$$\begin{array}{cc} & \frac{dv_{2m}}{dt}=\alpha_{m}\beta_{m}e^{-\mu \tau_{m}}s\left(t-\tau_{m}\right)v_{1m}\left(t-\tau_{m}\right)-\delta_{m}v_{2m}\left(t\right). \end{array}$$

(B1d)
$$\begin{array}{cc} & \frac{dv_{1}}{dt}=-\delta v_{1}\left(t\right), \end{array}$$

(B1e)
$$\begin{array}{cc} & \frac{dv_{2}}{dt}=\alpha \beta e^{-\mu \tau }s\left(t-\tau \right)v_{1}\left(t-\tau \right)-\delta v_{2}\left(t\right), \end{array}$$

with initial conditions: $s\left(0\right)=0,v_{1m}\left(0^{+}\right)=v_{2m}\left(0^{-}\right),v_{2m}\left(\tau \right)=0,v_{1}\left(0^{+}\right)=v_{2}\left(0^{-}\right),v_{2}\left(\tau \right)=0$. *m* subscripts refer to the invading mutant parasite and its corresponding traits.

Again, separate solutions for (B1a)–(B1c) are required depending on whether τ and $\tau_{m}$ are greater or less than $T-t_{l}$. The length of τ relative to *T* and $t_{l}$ determines ${\hat{v}}^{*}$, whereas the length of $\tau_{m}$ relative to *T* and $t_{l}$ determines the within‐season dynamics of the mutant parasite. The solutions to all cases can be found in the code on Github (see “Data Availability Statement” in the main text for the link). We first show the solution to the case when $\tau_{m}<T-t_{l}$:

$$\begin{array}{cc} & v_{1m}\left(t\right)=v_{1m}\left(0\right)e^{-\delta_{m}t}0<t<T\\ & s\left(t\right)=\left\{\begin{array}{cc} \frac{\hat{s}}{t_{l}}e^{\left(-\mu t+\frac{\alpha {\hat{v}}^{*}e^{-\delta t}}{\delta }+\frac{\alpha_{m}v_{1m}\left(0\right)e^{-\delta_{m}t}}{\delta_{m}}\right)}\int_{0}^{t}e^{\left(\mu u-\left(\frac{\alpha {\hat{v}}^{*}e^{-\delta u}}{\delta }+\frac{\alpha_{m}v_{1m}\left(0\right)e^{-\delta_{m}u}}{\delta_{m}}\right)\right)}du & 0<t<t_{l}\\ s\left(t_{l}\right)e^{(-\mu \left(t-t_{l}\right)-\left(\frac{(\alpha {\hat{v}}^{*}e^{-\delta (t-t_{l})}\left(-1+e^{\delta t}\right)}{\delta }+\frac{\alpha_{m}v_{1m}\left(0\right)e^{-\delta_{m}\left(t-t_{l}\right)}\left(-1+e^{\delta_{m}t}\right)}{\delta_{m}}\right)} & t_{l}\leq t<T \end{array}\right.\\ & v_{2m}\left(t\right)=\left\{\begin{array}{cc} \frac{\alpha_{m}\beta_{m}e^{-\mu \tau_{m}}v_{1m}\left(0\right)\hat{s}}{t_{l}}e^{-\delta_{m}\left(t-\tau_{m}\right)}\int_{0}^{t-\tau_{m}}e^{\left(-\mu u+\frac{\alpha {\hat{v}}^{*}e^{-\delta u}}{\delta }+\frac{\alpha_{m}v_{1m}\left(0\right)e^{-\delta_{m}u}}{\delta_{m}}\right)} & \\ \int_{0}^{u}e^{\left(\mu x-\left(\frac{\alpha {\hat{v}}^{*}e^{-\delta x}}{\delta }+\frac{\alpha_{m}v_{1m}\left(0\right)e^{-\delta_{m}x}}{\delta_{m}}\right)\right)}dxdu & \tau_{m}<t<t_{l}\\ e^{-\delta_{m}\left(t-t_{l}-\tau_{m}\right)}(v_{2}\left(t_{l}\right)+\alpha_{m}\beta_{m}e^{-\mu \tau_{m}}v_{1m}\left(0\right)s\left(t_{l}\right) & \\ \int_{0}^{t-t_{l}-\tau_{m}}e^{-\frac{\alpha_{m}v_{1m}\left(0\right)e^{-\delta_{m}\left(u+t_{l}\right)}\left(-1+e^{\delta_{m}u}\right)}{\delta_{m}}-\frac{\alpha {\hat{v}}^{*}e^{-\delta (u+t_{l})}\left(-1+e^{\delta u}\right)}{\delta }-\delta_{m}t_{l}-\mu u}du) & t_{l}\leq t\leq T. \end{array}\right. \end{array}$$

When $\tau_{m}>T-t_{l}$, the solution for $v_{2m}\left(t\right)$ is

$$\begin{array}{cc} v_{2m}\left(t\right)= & \frac{\alpha_{m}\beta_{m}e^{-\mu \tau_{m}}v_{1m}\left(0\right)\hat{s}}{t_{l}}e^{-\delta_{m}\left(t-\tau_{m}\right)}\int_{0}^{t-\tau_{m}}e^{\left(-\mu u+\frac{\alpha {\hat{v}}^{*}e^{-\delta u}}{\delta }+\frac{\alpha_{m}v_{1m}\left(0\right)e^{-\delta_{m}u}}{\delta_{m}}\right)}\\ & \int_{0}^{u}e^{\left(\mu x-\left(\frac{\alpha {\hat{v}}^{*}e^{-\delta x}}{\delta }+\frac{\alpha_{m}v_{1m}\left(0\right)e^{-\delta_{m}x}}{\delta_{m}}\right)\right)}dxdu\;\tau_{m}<t<T. \end{array}$$

The invasion fitness of a rare mutant parasite is given by the density of $v_{2m}$ produced by the end of the season. When $\tau_{m}<T-t_{l}$, the mutant parasite invades in a given host phenological scenario if the density of $v_{2m}$ produced by time *T* is greater than or equal to the initial $v_{1m}\left(0\right)=1$ introduced at the start of the season ($v_{2m}\left(T\right)\geq 1$), following

$$\begin{array}{cc} v_{2m}\left(T\right)= & e^{-\delta_{m}\left(T-t_{l}-\tau_{m}\right)}(v_{2}\left(t_{l}\right)+\alpha_{m}\beta_{m}e^{-\mu \tau_{m}}v_{1m}\left(0\right)s\left(t_{l}\right)\\ & \int_{0}^{T-t_{l}-\tau_{m}}e^{-\frac{\alpha_{m}v_{1m}\left(0\right)e^{-\delta_{m}\left(u+t_{l}\right)}\left(-1+e^{-\delta_{m}u}\right)}{\delta_{m}}-\frac{\alpha {\hat{v}}^{*}e^{-\delta (u+t_{l})}\left(-1+e^{\delta u}\right)}{\delta }-\delta_{m}t_{l}-\mu u}du). \end{array}$$

When $\tau_{m}>T-t_{l}$, the mutant parasite invades in a given host phenological scenario if the density of $v_{2m}$ produced by time *T* is greater than or equal to the initial $v_{1m}\left(0\right)=1$ introduced at the start of the season ($v_{2m}\left(T\right)\geq 1$), following

$$\begin{array}{cc} v_{2m}\left(T\right)= & \frac{\alpha_{m}\beta_{m}e^{-\mu \tau_{m}}v_{1m}\left(0\right)\hat{s}}{t_{l}}e^{-\delta_{m}\left(T-\tau_{m}\right)}\int_{0}^{T-\tau_{m}}e^{\left(-\mu u+\frac{\alpha {\hat{v}}^{*}e^{-\delta u}}{\delta }+\frac{\alpha_{m}v_{1m}\left(0\right)e^{-\delta_{m}u}}{\delta_{m}}\right)}\\ & \int_{0}^{u}e^{\left(\mu x-\left(\frac{\alpha {\hat{v}}^{*}e^{-\delta x}}{\delta }+\frac{\alpha_{m}v_{1m}\left(0\right)e^{-\delta_{m}x}}{\delta_{m}}\right)\right)}dxdu. \end{array}$$

We use $v_{2m}\left(T\right)$ to find optimal virulence for a given host phenological scenario by finding the trait value that maximizes $v_{2m}\left(T\right)$. That is, the virulence trait, $\tau^{*}$, that satisfies

(B2)
$$\begin{array}{cc} & \frac{dv_{2m}\left(T\right)}{d\tau_{m}}{|}_{\tau_{m}=\tau_{r}}=0 \end{array}$$

(B3)
$$\begin{array}{cc} & \frac{d^{2}v_{2m}\left(T\right)}{d\tau_{m}^{2}}{|}_{\tau_{m}=\tau_{r}}<0. \end{array}$$

For all phenological patterns, we found that $\tau^{*}$ is uninvadable, that is, condition (B3) is satisfied.

For certain phenological patterns, $\tau_{m}$ switches from $T-t_{l}<\tau_{m}$ to $T-t_{l}>\tau_{m}$ as it evolves. When $t_{l}$ is small, optimal virulence, $\tau^{*}$, is short relative to *T* and $t_{l}$. Thus when $t_{l}$ is short, $\tau^{*}$ times parasite‐induced death to begin *after* all hosts have finished emerging (i.e., the solution where $T-t_{l}>\tau_{m}$ is required to find $\tau^{*}$). For large $t_{l}$, the value of $\tau^{*}$ that optimizes parasite fitness initiates parasite‐induced host death *before* all hosts have finished emerging (*i.e*. the solution where $T-t_{l}<\tau_{m}$ is required to find $\tau^{*}$). We found the value of $t_{l}$ that requires a switch from the solution for $T-t_{l}>\tau_{m}$ to the solution for $T-t_{l}<\tau_{m}$ to find $\tau^{*}$ numerically using Mathematica. We switched which solution we used to find $\tau^{*}$ when the value of $\tau^{*}$ that satisfied (B2) no longer met the inequality. For example, for long $t_{l}$, the solution for $T-t_{l}>\tau_{m}$ returned $T-t_{l}<\tau^{*}$. When this occurred we switched to using the solution for $T-t_{l}<\tau_{m}$ to find $\tau^{*}$.

To study the impact of mechanistic trade‐offs between transmission and virulence on virulence evolution, we assume that the number of parasites produced at host death is a function of the time between infection and host death (β(τ)). This is done in Figure 3a,b where a mechanistic trade‐off is assumed to exist between τ and β in (B2). The same approach as described above is used to determine which solution correctly specifies $\tau^{*}$.
